# Antibody reaction of leptospirosis in asymptomatic feral boars, Thailand

**DOI:** 10.14202/vetworld.2019.1884-1887

**Published:** 2019-11-29

**Authors:** Phirom Prompiram, Kanaporn Poltep, Nongluck Sangkaew

**Affiliations:** 1 The Monitoring and Surveillance Center for Zoonotic Diseases in Wildlife and Exotic Animals, Faculty of Veterinary Science, Mahidol University, 999 Phuttamonthon 4 Rd., Salaya, Phutthamonthon, Nakhon Pathom 73170, Thailand; 2 Veterinary Research and Development Center Lower Northern Region, 9 Moo15 Phitsanulok-Lomsak Rd., Wangthong, Phitsanulok 65130, Thailand

**Keywords:** Ballum serovar, Canicola serovar, feral boar, microscopic agglutination test, Thailand

## Abstract

**Aims:**

This study aimed to determine the proportion of exposure to leptospirosis and evaluate the degree of serovar antibody reaction in feral boars.

**Materials and Methods:**

A total of 58 sera obtained from feral boars in Khao Prathab Chang Wildlife Breeding Center, Ratchaburi, Thailand, were screened for leptospirosis exposure by microscopic agglutination test, conducted with a reference panel of 23 pathogenic serovars and a non-pathogenic serovar.

**Results:**

Overall exposure rate of 62.07% was found in the studied population. An antibody reaction presented in 18 of 24 leptospiral serovars. Among the seropositive, Ballum serovar showed predominant exposure in the feral boar population.

**Conclusion:**

The results show a relatively high exposure to leptospirosis and the predominant serovar was Ballum followed by Canicola, the first finding in feral boars in Thailand. It has been revealed that feral boars act as a natural reservoir host of leptospirosis. There should be more concern about public health problems in leptospirosis arising where feral boars appear.

## Introduction

Leptospirosis, a bacterial zoonotic disease, has shown a spatiotemporal incidence in the peoples of tropical regions with high temperature and humidity, including Thailand. During January-February 2019, one individual died among 186 cases of leptospirosis reported from 42 provinces around Thailand [1]. Moreover, the incidence of leptospirosis in Thailand is commonly involved with wildlife species, which are an important source of pathogenic serovars [2,3].

The role of wildlife in harboring leptospirosis first focused on rodent species; however, several species of wildlife, including primate species, have been shown significantly in harboring leptospirosis [3,4]. To better understand the reservoir hosts or facilitating hosts of leptospirosis exposure in other wildlife, in particular, the feral boar should be of concern as a natural reservoir host, because of its feeding behavior on the ground that a leptospirosis endemic source, and they are considered highly mobile animals [5].

In the previous reports from both America and continental Europe, feral boars have been seen to harbor various predominant serovars of leptospirosis [6-9]. If feral boars are exposed to the various leptospiral serovars, it could be another reservoir which could indicate the predominant serovar that might be of public significance.

Therefore, this study aimed to determine the extent of exposure to leptospirosis and to evaluate the predominant serovar in feral boars for the 1^st^ time of Thailand.

## Materials and Methods

### Ethical approval

The procedures of restraint and blood collection for the feral boars were approved by the Mahidol University Application for Permission for Animal Care and Use: MUVS-2009-05, Faculty of Veterinary Science, Mahidol University, Thailand.

### Study site

Feral boars were collected inside Khao Prathab Chang Wildlife Breeding Center, Ratchaburi, which is located in the western part of Thailand. This center was originally an animal shelter that contained captive wildlife, including conserved primates, tigers, bears, deer, and several species of wild bird and feral animals, particularly free-ranging rodents and boar. Feral boar was first an illegal animal seized and then inbreeding to increase the population into about 200-250 individuals that were distributing inside the restricted area, approximately 900 acres around the captive animal area. Serum of asymptomatic feral boar was submitted by 58 individuals. All serum collections had been stored using cryopreservation (−80°C) at the Faculty of Veterinary Science, Mahidol University, since 2009 until laboratory testing.

### Microscopic agglutination test (MAT)

At the Veterinary Research and Development Center, Northern Lower Zone (Phitsanulok, Thailand), MAT was following the Manual of Diagnostic Tests and Vaccines for Terrestrial Animals for leptospirosis [10] with a reference panel of 23 pathogenic serovars and a non-pathogenic serovar, Patoc was performed. During the screening test, individual serum was diluted in phosphate-buffered saline and an equal volume of antigen was added by the final concentration was 1:100 following OIE recommendation and then, the mixtures were incubated at 30°C for 1.5 h. The antibody reactions were examined using the dark-field microscope at least 50% agglutination with each antigen considerable as the endpoint. The sample was showing the reaction for at least one serovar, considerable the seropositive for specific serovar. The significant of the test was determined by the Chi-square test, at p<0.05.

## Results and Discussion

This study is a preliminary documented the leptospirosis in feral boar of Thailand. Khao Prathab Chang Wildlife Breeding Center was established for conservative wildlife and resting the illegal animal with various species of wildlife. Among animal species, feral boar is one significant species in this area due to widespread and close to natural environment. To determine the proportion of leptospirosis exposure in feral boar, MAT was performed to detect the reactive antibody of leptospirosis. It is significant to note that the present study used the endpoint titer of 1:100, a single dilution as recommended by OIE [10] for screening test, although, the positive was set at titer of equal or more than 1:100. However, other study in livestock of Thailand, the positive by MAT was considered at less than dilution for the titer of equal or more than 1:50 [11]. Therefore, the proportion of leptospire exposure in this study is likely underestimated due to some feral boar that was positive reaction at less dilution not tested.

The overall exposure rate of the asymptomatic feral boars here was 36 of 58 (62.07%; 95% confidence interval [CI] 60.94-63.20). This is considered a high exposure rate among wildlife species [3,4]. It certainly demonstrates a natural harboring source of the leptospiral bacteria in feral boars. From the previous studies in feral boars, the exposure rate is different when considering the different geographic location or the reference panel of antigens used [6,8,9,12]. However, a high exposure rate is further associated with host susceptibility since feral boars live close to a contaminated environment, so there is the possibility of exposure to leptospires throughout their lifespan with a wide range distribution [5,13]. This study supports the role of a natural reservoir host of leptospirosis showing 36 feral boars which were found seropositive with 18 of 24 serovars (Table-1). They were infected naturally and reflect the serovars circulating in the geographic habitat. From the different geographic areas of a previous study in feral boars, a variety of predominant serovars, including Bratislava (serogroup Australis), Pomona, and Tarassovi were found, and this may reflect the specificity of an individual area. In addition, a panel of non-pathogenic serovars using Patoc was tested. It was rarely reactive, due to non-specific immune defense, and the test was completely clear. Nevertheless, infection is likely to be found in a resistance incompetent host or with a high level of organism load that results in finding reactive antibodies. This study consistently found that the test was negative to Patoc, agreeing with the study using MAT in Thai livestock which found seronegativity among buffaloes, sheep, and goats [11]. However, Patoc is usually used to develop the agglutination test to screen for leptospire infection showing less specificity than MAT, a gold standard for detecting immune response to leptospirosis that was performed in this study.

**Table-1:** The overall seropositive by using microscopic agglutination test (MAT) in 36 out of 58 wild boars from Khao Prathab Chang Wildlife Breeding Center, Thailand.

Serovar*	n (seropositive)	Exposure rate (%)	95%CI	

			lower	upper
Ballum	18	31.03	29.90	32.17
Canicola	13	22.41	21.28	23.55
Djasiman	9	15.52	14.39	16.65
Shermani	8	13.79	12.66	14.93
Cynopteri	6	10.34	9.21	11.48
Bratislava	5	8.62	7.49	9.75
Hebdomadis	5	8.62	7.49	9.75
Ranarum	5	8.62	7.49	9.75
Grippotyphosa	4	6.90	5.76	8.03
Javanica	4	6.90	5.76	8.03
Autumnalis	3	5.17	4.04	6.30
Bataviae	3	5.17	4.04	6.30
Celledoni	3	5.17	4.04	6.30
Manhao	3	5.17	4.04	6.30
Mini	3	5.17	4.04	6.30
Pomona	3	5.17	4.04	6.30
Pyrogenes	1	1.72	0.59	2.86
Tarassovi	1	1.72	0.59	2.86

* The negative response found the antibody to Icterohaemorrhagiae, Louisiana, Panama, Patoc, Sarmin and Sejroe

In these study findings (Table-1), the predominant leptospirosis exposure (n=18) higher than others (p≤0.05) was the Ballum serovar with 31.03% (95% CI 29.90-32.17), showing a difference from serovars found in other feral boars from the previous studies and moreover different from that found in the pig farms of Thailand, which is the Ramarum serovar [11,14]. This finding is the first report of the predominant serovar being Ballum in feral boars and furthermore in the country of Thailand. In the previous studies, the Ballum serovar has been isolated from the African giant pouched rat (*Cricetomys gambianus*) urine and is frequently found as associate evidence with the positive reaction antibody in humans [15,16]. It reveals that Ballum, the predominant serovar of leptospirosis found in this study, has been a significant serovar either harboring the chain of infection with rodent species or having an effect on public health problems. Moreover, the serovar with a high antibody reaction (n=13, Table-1) was the Canicola serovar with 22.41% (95% CI 21.28-23.55). The previous study has shown Canicola serovar typing by pulsed-field gel electrophoresis, variable number tandem repeat analysis, and single-enzyme amplified fragment length polymorphism in canines and porcine that were identical with bovines [17]. However, this serovar is usually found to have more exposure in canines than porcine with feral boars as a member, and moreover, Canicola serovar can be found in various animal reservoirs and humans, as implicated by antibody detection [18]. It indicates that possibly the host range of Canicola serovar has several animal reservoir hosts and causes pathogenicity in humans, despite one source being harbored by either canine or porcine.

Therefore, leptospirosis should be a concern in specific areas where feral boars appear to be a source of a zoonosis. Although the impact of the health problem on the public in the chain of infection is restrictedly needed by an individual serovar, it is possibly transmitted by other animal species such as companion animals or rats, which are more closely related to humans than feral boars. However, this study supports that feral boars play a role by naturally harboring the pathogenic serovars for leptospirosis, despite the predominant serovars depending on an individual area of the feral boar habitat.

## Conclusion

From this study, feral boars were seen to have high leptospiral exposure, confirming the source of various serovars representing the predominant, depending on the circulation of individual serovars in their habitat. The knowledge of serovars in feral boars is the baseline for further awareness, preparedness, and prevention of leptospirosis in public. More details are needed about the relative incidence of leptospirosis in the public and feral boars.

## Authors’ Contributions

PP and KP collected and stored the feral boar serum. NS assisted in conducting the MAT. PP and KP partially scientifically analyzed the result, drafted, and revised the manuscript. All authors read and approved the final manuscript.
